# Mental health of older adults during the COVID-19 pandemic: lessons from history to guide our future

**DOI:** 10.1017/S1041610220001003

**Published:** 2020-06-03

**Authors:** Terence W. H. Chong, Eleanor Curran, David Ames, Nicola T. Lautenschlager, David J. Castle

**Affiliations:** 1 St Vincent’s Hospital Melbourne, Department of Psychiatry, The University of Melbourne, Parkville, Australia; 2 St Vincent’s Hospital Melbourne, Fitzroy, Australia; 3 Academic Unit for Psychiatry of Old Age, Department of Psychiatry, The University of Melbourne, Parkville, Australia; 4 North Western Mental Health, Melbourne Health, Parkville, Australia

**Keywords:** COVID-19, mental health, older adults, dementia, nursing homes, anxiety

Older adults have been identified as a “population of interest” by the recent *Lancet Psychiatry* position paper on multidisciplinary research priorities for the coronavirus disease 2019 (COVID-19) pandemic (Holmes *et al.*, 2020). The authors discuss issues that include “isolation, loneliness, end of life care, and bereavement,” noting that these may be exacerbated by the “digital divide” (Holmes *et al*., 2020). Psychological stress in older adults stem from not only excess mortality risk leading many to self-isolate, but also restrictions instituted to mitigate COVID-19 risk. This is highlighted by a recent review of the psychological impact of quarantine that identified an increase in post-traumatic stress symptoms, confusion, and anger (Brooks *et al*., 2020).

Learning from history is a helpful starting point. Even as far back as the 1918–1919 “Spanish” influenza pandemic, there has been discussion of an increase in rate of psychosis and suicide at that time (Harris, 2006). The impact of the Severe Acute Respiratory Syndrome (SARS) epidemic in 2003 on the mental health of older adults has received some attention. Compared with 2002 (28.4 per 100,000), the suicide rate in older adults in Hong Kong was higher in 2003 (40.4 per 100,000) and 2004 (34.0 per 100,000) (Cheung *et al*., 2008). It was postulated that loneliness and disconnectedness were associated with this increase. The prevalence of probable post-traumatic stress disorder in Hong Kong was associated with older age and residing in SARS-prevalent regions (Lee *et al*., 2006).

The very recent history of providing mental health services to older adults in China during the COVID-19 pandemic has been challenging, with the “digital divide,” quarantines, and restrictions on movement having significant impact (Yang *et al*., 2020). Many older adults have experienced reduced access to specialist services and hospitals and delayed presenting for medical care due to fear of contracting COVID-19. Nursing homes have experienced staff and personal protective equipment shortages, and their residents have comprised a significant proportion of COVID-19 mortality (Pachana *et al*., 2020). Residents have experienced relapse of their mental health condition due to restrictions on visitors and limited access to technology to facilitate other types of contact; a phone call or wave through the window is just not the same. However, many patients have embraced interventions delivered using technology such as telehealth, particularly when provided with support.

Older adults living with dementia in the COVID-19 world have experienced reduced access to support and activities. These changes have caused distress and exacerbated behavioral and psychological symptoms of dementia. We have seen community-dwelling older adults with dementia referred due to inability to understand and retain information about social distancing, with relatives fearing they will be at risk of contracting COVID-19 or receiving penalties from law enforcement agencies. A concerning development is the increasing media discussion about the relative worth of human lives based on age and remaining life expectancy which could divide generations and challenge established ethical values.

Our response to COVID-19 for older adults can borrow general mental health recommendations from historic pandemic influenza preparedness initiatives including a focus on strengthening social networks and community support, communication networks, directive leadership and stress management (Douglas *et al*., 2009). One of the few positives in the pandemic literature in older adults is the possibility that older adults may experience more adaptive coping in a crisis than younger adults, as suggested by a study in Hong Kong during the SARS epidemic (Yeung *et al*., 2007). This resilience may relate to their lived experience of coping with adversity and could potentially be harnessed now.

It is heartening to see the relative increase in attention being paid to mental health by governments, researchers, and clinicians for those affected by COVID-19, when compared to previous pandemics (*Lancet Psychiatry*, 2020). Targeted research will be critical to inform the optimization of older adults’ mental health in the face of COVID-19. It will be important to review the mental health effects of past pandemics and interventions that are effective. Additionally, we can learn from countries that have experienced the effects of COVID-19 earlier. Unfortunately, research into the mental health of older adults has been affected by social distancing, nursing home restrictions, and an overwhelmed health system. Given that the impact of this pandemic may be protracted, clinicians and researchers will need to explore novel ways to reach older adults and provide interventions to improve their mental health during COVID-19 and into the future.
